# Clinicopathological Characteristics and Prognosis of cT1N0M1 Gastric Cancer: A Population-Based Study

**DOI:** 10.1155/2019/5902091

**Published:** 2019-05-02

**Authors:** Jianbo Han, Junhao Tu, Chaoyang Tang, Xiang Ma, Chi Huang

**Affiliations:** ^1^Department of General Surgery, Nanjing Red Cross Hospital, Nanjing, 210001 Jiangsu Province, China; ^2^Department of General Surgery, Suzhou Wuzhong People's Hospital, Suzhou, 215128 Jiangsu Province, China; ^3^Department of General Surgery, The Second Affiliated Hospital of Nanjing Medical University, Nanjing, 210011 Jiangsu Province, China; ^4^Department of General Surgery, Affiliated Hospital of Integrated Chinese and Western Medicine, Nanjing University of Chinese Medicine, Nanjing, 210028 Jiangsu Province, China

## Abstract

**Background:**

Distant metastasis of early gastric cancer is a rare subgroup and poorly understood. The present study is aimed at summarizing the clinicopathological characteristics, prognosis, and management of clinical T1N0M1 (cT1N0M1) gastric cancer.

**Method:**

Between 2004 and 2015, patients diagnosed with cT1N0M1 gastric cancer were retrospectively analyzed using the Surveillance, Epidemiology, and End Results (SEER) database.

**Results:**

A total of 1093 cT1N0M1 gastric cancer patients were identified. 49 patients (4.5%) received cancer-directed surgery, and 113 patients (10.4%) were managed with radiotherapy. Compared with the other stage IV diseases, a relatively high proportion of black population (19.9% vs. 15.8%), patients older than 60 years (63.1% vs. 57.8%), and adenocarcinoma (59.5% vs. 55.9%) were observed in the cT1N0M1 gastric cancer subgroup. Besides that, patients with cT1N0M1 had the characteristics of less poor differentiated or undifferentiated (54.3% vs. 61.7%). Patients with cT1N0M1 had worse cancer-specific survival (CSS) and overall survival (OS) as compared to the other metastatic gastric cancer patients (CSS: *p* = 0.002, OS: *p* = 0.001 for log-rank test). Intriguingly, patients with cT1N0M1 had poor prognosis as compared to patients with cT1N+M1 (CSS: *p* = 0.015, OS: *p* = 0.007 for log-rank test). The 3-year and 5-year CSS for patients with cT1N0M1 were 5.7% and 4.0%, respectively. The addition of surgery resulted in improved CSS (*p* < 0.001 for log-rank test) while radiotherapy was not associated with CSS (*p* = 0.756 for log-rank test) in patients with cT1N0M1. A multivariate Cox analysis showed that surgery (HR = 0.378, 95% CI: 0.255-0.562) and patients younger than 60 (HR = 0.745, 95% CI: 0.647-0.858) years were independent protective factors for these subgroup patients.

**Conclusion:**

Patients with cT1N0M1 gastric cancer had distinctive clinicopathological characteristics and presented poor prognosis. Knowledge of these differences contributes to guiding clinical evaluation for metastatic gastric cancer patients. More aggressive therapeutic strategy should be highlighted for this subgroup.

## 1. Introduction

Gastric cancer (GC) is one of the most common cancers worldwide and is responsible for over 1,000,000 new diagnosed cases and an estimated 783,000 deaths in 2018. Besides that, GC remains the third leading cause of cancer mortality [1]. Systemic treatment of GC has remarkably improved the long-term survival of GC patients, especially in early ones, which 5-year survival rate can reach more than 90% [2, 3]. However, the overall prognosis of advanced GC remains very poor, especially in stage IV GC patients with the 5-year survival rate being about 10% [4].

General prognostic factors of GC including depth of wall invasion, lymph node or distant metastasis status, age, and genetic factors have been well recognized [5–7]. Nomograms were built and validated on the basis of prognostic factors for predicting the overall survival or disease-free survival in different GC subgroups with guiding optimal therapy [8–10]. Normally, the deeper the tumor infiltrates and the more lymph node metastasizes, the worse the prognosis of GC patients is. A previous study reported that the survival rates of patients with pT1, pT2, pT3, and pT4 stage tumors were 89.3%, 72.4%, 36.9%, and 23.7%, respectively [5]. And higher lymph node ratios are significantly associated with a shorter overall survival [6]. According to the 8th AJCC staging system, stage IV includes the TanyNanyM1, T1-3N3M0, T4N1-2M0, and T4N3M0 groups. A previous study reported that the survival rate of patients with subclassification IVa gastric cancers was significantly higher than that of patients with subclassification IVb ones [11].

The diagnostic rate of early GC (EGC) has increased in recent years, possibly due to a combination of increased screening and improved diagnostic techniques. EGC is defined as lesion confined to the mucosa and submucosa regardless of status of lymph node with favorable prognosis [12]. Very rare cases of EGC developed to distant metastasis (T1N_X_M1). But the prognosis of EGC with distant metastasis is poor defined because of the limited cases. Most people may think that T1N0M1 GC patients with mild gastric wall invasion should have better prognosis than T1NanyM1 and other M1 (T2-4NanyM1) patients. Is it justified? In order to address this question, in the present study, we delineated clinicopathological characteristics and prognosis of clinical T1N0M1 (cT1N0M1) gastric cancer using the Surveillance, Epidemiology, and End Results (SEER) database, to develop a clinicopathological risk score that can be used preoperatively to determine the risk of cT1N0M1 patients.

## 2. Material and Methods

### 2.1. Data Collection

Patients with metastatic GC were included from the SEER database (2004-2015). Of these, 1093 patients presented stage cT1N0M1. This study was approved by the Institutional Review Board of the Second Affiliated Hospital of Nanjing Medical University. The inclusion criteria were summarized as follows: the site code represented “stomach (143),” patients with distant metastases (M1) according to American Joint Committee on Cancer 7th edition, GC was diagnosed by positive histology or cytology, GC was the only type of primary cancer, and information about cancer-specific survival (CSS) and overall survival (OS) months was clear.

The following data were extracted: gender, age at diagnosis, marital status, race, histologic type, differentiation status, T stage, N stage, surgery, radiation, survival months, CSS, and OS. CSS was defined as the time from the date of diagnosis to the date of death caused by GC.

### 2.2. Statistical Analysis

The differences between groups were determined by using the *χ*^2^ test. The Kaplan-Meier method was utilized to analyze CSS and OS. The difference was identified with log-rank test. Multivariate analyses were performed to recognize the independent prognostic factors for CSS. All statistical analyses were performed with SPSS 25.0.

## 3. Results

### 3.1. Characteristics of Patients

A total of 13,253 metastatic GC patients were identified. 1093 patients were diagnosed at stage cT1N0M1. In this subgroup, 49 (4.5%) received cancer-directed surgery and 113 (10.4%) were managed with radiotherapy. 351 patients with records of definite organ metastases were available. 291 patients suffered isolated organ involvement and 60 patients experienced multiple organ metastases. The most commonly single involved site is the liver (53%), followed by the bone (16.8%), the lung (12.3%), and the brain (0.9%). Compared with the other stage IV diseases, a relatively high proportion of black population (19.9% vs. 15.8%), patients older than 60 years (63.1% vs. 57.8%), and signet ring cell carcinoma (59.5% vs. 55.9%) were observed in the cT1N0M1 GC subgroup. Besides that, patients with cT1N0M1 had the characteristics of less poor differentiated or undifferentiated (54.3% vs. 61.7%). No differences were observed in terms of marital status, sex, and metastatic sites. The details are summarized in Table 1.

### 3.2. Survival Outcomes

**Figure 1:**
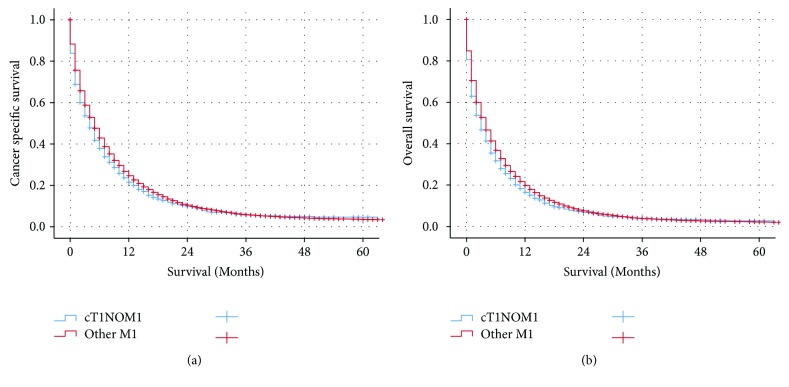
The Kaplan-Meier survival curves revealed that patients with cT1N0M1 had worse cancer-specific survival and overall survival as compared to the other metastatic gastric cancer patients.

**Figure 2:**
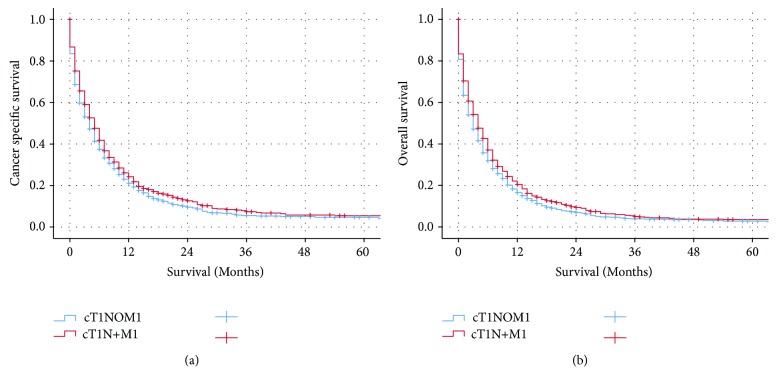
The Kaplan-Meier survival curves revealed that patients with cT1N0M1 had worse cancer-specific survival and overall survival as compared to patients with cT1N+M1.

**Figure 3:**
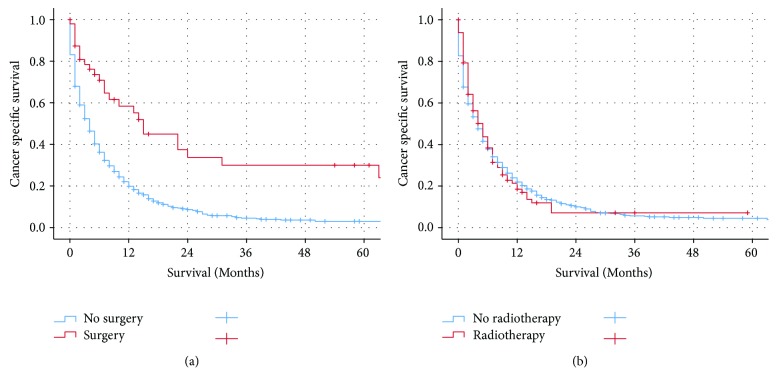
The Kaplan-Meier survival curves revealed that the addition of surgery resulted in improved CSS while radiotherapy was not associated with cancer-specific survival in patients with cT1N0M1.

Median CSS for GC patients with stage cT1N0M1, the other stage IV patients, and cT1N+M1 were 4, 5, and 5 months, respectively. Patients with cT1N0M1 had worse cancer-specific survival (CSS) and overall survival (OS) as compared to the other metastatic GC patients (CSS: *p* = 0.002, OS: *p* = 0.001 for log-rank test) (Figure 1). Besides that, patients with cT1N0M1 had poor prognosis as compared to patients with cT1N+M1 (CSS: *p* = 0.015, OS: *p* = 0.007 for log-rank test) (Figure 2). The 3-year and 5-year CSS for patients with cT1N0M1 were 5.7% and 4.0%, respectively. The 3-year and 5-year CSS for patients with other M1 were 5.8% and 3.5%, respectively. The 3-year and 5-year CSS for patients with cT1N+M1 were 7.4% and 4.9%, respectively. The addition of surgery resulted in improved CSS (*p* < 0.001 for log-rank test) while radiotherapy was not associated with CSS (*p* = 0.756 for log-rank test) in GC patients with cT1N0M1 (Figure 3).

In Cox multivariate regression analysis, surgery (HR = 0.378, 95% CI: 0.255-0.562) and patients younger than 60 years (HR = 0.745, 95% CI: 0.647-0.858) were independent protective factors for this subgroup patients (Table 2).

## 4. Discussion

Over the past decades, risk factors of lymph node metastasis in EGC have been well established and scholars have reached consensus on endoscopic submucosal dissection and endoscopic mucosal resection for EGC [13–16]. However, EGC with distant metastasis has been rarely described. Only scattered case reports presented the limited characteristics of this rare situation, and the incidence is about 0.14% [17–20]. To our knowledge, this is the first retrospective study reported clinicopathological characteristics and prognosis of cT1N0M1 GC with large sample size.

Our study demonstrated that surgery improved the prognosis of cT1N0M1 GC patients while radiotherapy did not. This rare entity is consistent with the general stage IV GC in terms of palliative surgery [21]. A previous review has interpreted the survival benefit of gastrectomy compared to nonoperative treatment for stage IV GC [22]. For cT1N0M1 GC, surgery should be taken into account in a proper way. Further studies are needed to establish optimized regimes for the management of this rare entity.

The classic progressive pattern of GC refers to spreading to nearby tissues and perigastric or distant lymph nodes and metastasizing to distant organs. GC seldom presents distant metastases within stage T3. Our study demonstrated that GC patients with cT1N0M1 had worse prognosis as compared to the other stage IV GC patients including stage cT1N+M1. cT1N0M1 GC skipped lymph node involved and directly metastasized to distant organs. We hypothesized that this subgroup is associated with more aggressive tumor behaviors and predicts poor prognosis. Similarly, compared with the other metastatic GC, tumors with mild gastric wall invasion and negative lymph nodes represent more aggressive malignancies with a distinct biology. Unexpectedly, a relatively less proportion of signet ring cell carcinoma and more well-differentiated patients in the cT1N0M1 GC subgroup complicated this rare entity. Precision mechanisms and distinct biology merit further investigation. Collectively, this rare entity requires more intensive intervention and follow-up due to dismal prognosis.

In our present study, the proportion of EGC distant metastasis seems to be higher than previous literature reports [23]. Only 49 (4.5%) patients received cancer-directed surgery with clear pathological outcomes, and the others were diagnosed with clinical stage. We speculate that ultrasound gastroscopy and CT as the main clinical stages for GC may underestimate the clinical T stage. Some patients with stage T2 are often mistaken for stage T1 [24]. However, it remains true that tumors with mild gastric wall invasion metastasizing to distant organs predicted an extremely poor prognosis.

Additionally, there are several limitations in our study. Firstly, stage IV consists of heterogeneous subgroups including TanyNanyM1, T1-3N3M0, T4N1-2M0, and T4N3M0. In the present study, we only compared the prognosis of cT1N0M1 with other stage IV ones (cT1N+M1 and cT2-4NanyM1), but not with T1-3N3M0, T4N1-2M0, and T4N3M0, respectively. Secondly, as mentioned above, the clinical TNM stage of GC patients was determined by imaging results with a gap compared with the pathological TNM stage. The evidences for the diagnosis of distant metastasis are sometimes insufficient. Finally, the study was designed based on the condition of USA population, and the conclusions should thus be extended to other ethnic groups with caution.

In conclusion, we first evaluated clinicopathological characteristics and prognosis of cT1N0M1 GC with large sample size. Our results showed that GC patients with cT1N0M1 had worse CSS and OS as compared to the other M1 GC patients, and patients with cT1N0M1 had poor prognosis as compared to patients with cT1N+M1. Sometimes small tumors go big. Knowledge of these differences is conducive to guiding clinical evaluation for metastatic GC patients and highlighting more aggressive therapeutic strategy.

## Figures and Tables

**Table 1:** Clinical characteristics of patients with metastatic gastric cancer according to different clinical stages.

Variable	cT1N0M1		Other M1		*p* value
	Number	%	Number	%	
Age					0.001
>60	690	63.1	7032	57.8	
≤60	403	36.9	5128	42.2	
Sex					0.262
Male	603	55.2	6922	56.9	
Female	490	44.8	5238	43.1	
Marital status					0.269
Unmarried	464	42.5	4878	40.1	
Married	582	53.2	6784	55.8	
Unknown	47	4.3	498	4.1	
Race					<0.001
White	719	65.8	8015	65.9	
Black	217	19.9	1919	15.8	
Others	157	14.4	2226	18.3	
Histologic type					0.026
Adenocarcinoma	650	59.5	6803	55.9	
Signet ring cell carcinoma	295	27.0	3369	27.7	
Others	148	13.5	1988	16.3	
Differentiation					<0.001
Well and moderate	231	21.1	1768	14.5	
Poor and undifferentiated	593	54.3	7499	61.7	
Unknown	269	24.6	2893	23.8	
Metastatic site					0.193
Bone	59	16.8	400	13.4	
Brain	3	0.9	35	1.2	
Liver	186	53.0	1705	57.3	
Lung	43	12.3	291	9.8	
Multiple organs	60	17.1	547	18.4	

**Table 2:** Univariate and multivariate analysis for cancer-specific survival in cT1N0M1 gastric cancer.

Variable	Univariate analysis		Multivariate analysis	
	Median survival time (month)	*p* value	HR (95% CI)	*p* value
Age		<0.001		
>60	3		Reference	
≤60	6		0.745 (0.647-0.858)	<0.001
Sex		0.179	NI	
Male	4			
Female	4			
Marital status		0.034	NI	
Unmarried	3			
Married	5			
Unknown	/		/	
Race		0.011	NI	
White	4			
Black	4			
Others	/			
Histologic type		0.221	NI	
Adenocarcinoma	4			
Signet ring cell carcinoma	4			
Others	6			
Differentiation		0.308	NI	
Well and moderate	4			
Poor and undifferentiated	4			
Unknown	/		/	
Surgery		<0.001		
No/unknown	4		Reference	
Yes	15		0.378 (0.255-0.562)	<0.001
Radiation		0.960	NI	
No/unknown	4			
Yes	4			

NI: not included in multivariate survival analysis.

## Data Availability

The datasets are available in the SEER repository and can be obtained from https://seer.cancer.gov.
